# Antimicrobial Treatment of *Serratia marcescens* Invasive Infections: Systematic Review

**DOI:** 10.3390/antibiotics12020367

**Published:** 2023-02-09

**Authors:** Radica Zivkovic Zaric, Milan Zaric, Marija Sekulic, Nenad Zornic, Jelena Nesic, Vesna Rosic, Tatjana Vulovic, Marko Spasic, Marko Vuleta, Jovan Jovanovic, Dalibor Jovanovic, Stefan Jakovljevic, Petar Canovic

**Affiliations:** 1 Faculty of Medical Sciences, University of Kragujevac, 34000 Kragujevac, Serbia; 2 Clinical Center Kragujevac, 34000 Kragujevac, Serbia; 3 General Hospital “Dragisa Misovic”, 11000 Belgrade, Serbia

**Keywords:** *Serratia marcescens*, invasive infection, antibiotics

## Abstract

*Background*: *Serratia marcescens* (SM) is a Gram-negative pathogen discovered by Italian pharmacist, Bizio, in 1819. According to the literature, *S. marcescens* is resistant to a wide range of antibiotics, including penicillin, cephalosporin, tetracycline, macrolide, nitrofurantoin, and colistin. We conducted a systematic review of published reports, determined what invasive infections could cause SM, and established the most appropriate antibiotic therapy. *Methods*: We registered this systematic review on the PROSPERO registry of systematic reviews–meta-analyses before we started our research (registration number CRD42022323159). The online searches of published studies were implemented via MEDLINE, the Cochrane Central Register of Controlled Trials, EBSCO, Scopus, Google Scholar, SCIndex, and the registry of clinical studies of human participants (ClinicalTrials.gov). *Results*: Our study included 32 published articles (9 case series and 23 case reports). There were 57 individual cases, respectively. The oldest patient was 97 years and the youngest patient was a newborn. *S. marcescens* was, in most cases, isolated from blood followed by urine and cerebrospinal fluid. In most cases, sensitivity was tested to cotrimoxazole (from 27 isolates, 10 showed resistance) followed by gentamicin (from 26 isolates, 3 showed resistance) as well as amikacin (from 21 isolates, none showed resistance). Patients died from an infection in 21 cases (31%). *Conclusions*: Treatment of SM infections should include carbapenems or aminoglycosides in combination with third-generation (and eventually fourth-generation) cephalosporin. Cotrimoxazole should be considered in cases of uncomplicated urinary infections.

## 1. Introduction

*Serratia marcescens* (SM), a Gram-negative pathogen, belongs to the genus Serratia and the Yersiniaceae family. It was discovered by Bartolomeo Bizio in 1819. Bizio, an Italian pharmacist and later a professor at the University of Padua, Italy, reported a blood-like pigmentation on polenta and rice. He classified the microorganism as a fungus and named it *S. marcescens*. Serratia was chosen in honor of Serafino Serrati, a physicist who ran the first steamboat on the Arno River in 1787, while marcescens comes from the Latin word “to decay” [1,2,3]. SM has the ability to produce red-pigmented prodigiosin [4]. Nowadays, no pigmented strains of SM are predominant over pigmented strains, especially in compromised patients. *Serratia marcescens* is usually isolated from blood and cultivated on blood agar or with selective culture methods (MacConcey or chromogenic agars) [4].

SM is usually resistant to antibiotics by producing deoxyribonuclease (DNase), lipase, and gelatinase [4]. *S. marcescens* also produces a pore-forming hemolysin, ShIA, which can cause cell cytotoxicity and the release of inflammatory mediators [5]. *Serratia marcescens* causes both opportunistic and nosocomial infections as well as a wide range of infections in both adults and children. The urinary catheter is thought to be the main entry point for infections, such as intubation and the central venous catheter [4]. Catheter colonization increases bacterial survival, especially in biofilm communities. Biofilm also increases resistance to antibiotics because it prevents their penetration [6]. Different types of infection by SM were described, such as pneumonia, sepsis, wound infection, meningitis, endocarditis, ocular infections, etc. [7,8,9,10].

SM, along with the *Enterobacter* spp., *Citrobacter freundii*, *Providencia* spp., and *Morganella morganii*, makes up the ‘ESCPM’ group and may express high levels of AmpC [11]. According to the literature, *S. marcescens* is resistant to a wide range of antibiotics, including penicillin, cephalosporin, tetracycline, macrolide, nitrofurantoin, and colistin. In the past, antibiotics, such as fluoroquinolones, aminoglycosides, and third-generation cephalosporins, were the bases for the treatment of *S. marcescens* infections. However, many clinical isolates of *S. marcescens* now show multiple forms of antimicrobial resistance to these antibiotics [4].

Guidelines for the treatment of *Serratia marcescens* infections do not exist, mostly because the relevant publications were based on individual case reports. We conducted a systematic review of the published reports and determined what invasive infections could be caused by SM, as well as established what the most appropriate antibiotic therapy is.

## 2. Results

**Figure 1:**
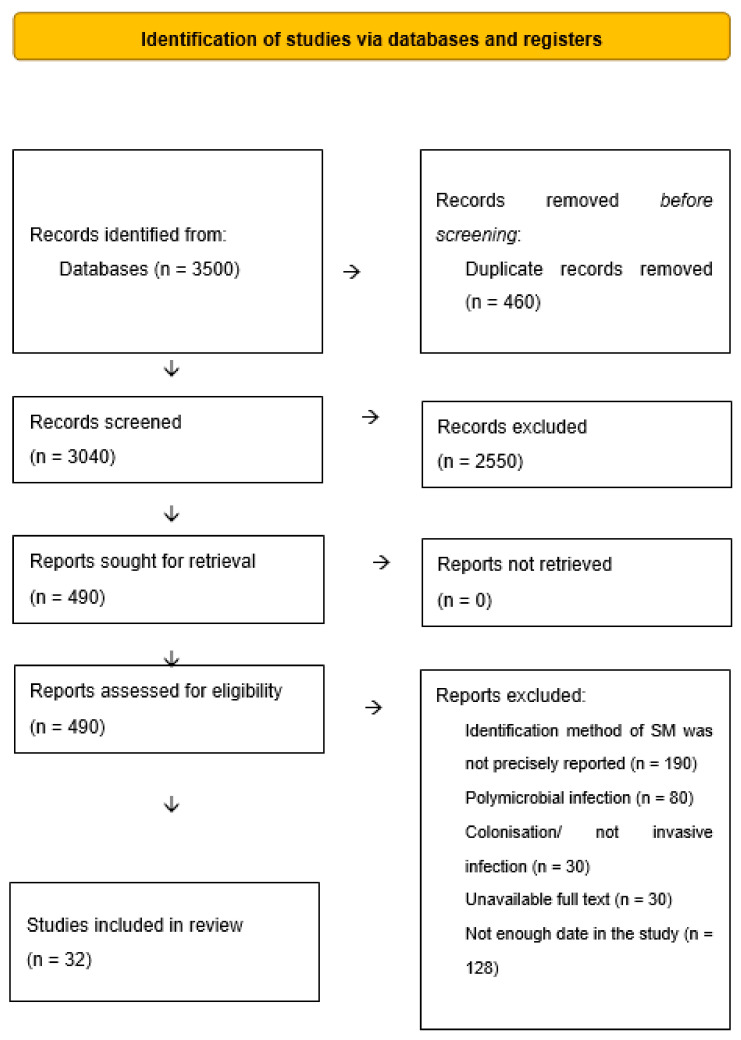
Selection of the studies.

Our study included 32 published articles (9 case series and 23 case reports) (Figure 1). There were 57 individual cases, respectively. The oldest patient was 97 years old and the youngest patient was a newborn. As for the age distribution of the patients, there were 3 (5%) newborns (<28 days), 8 (14%) infants (1 month–1 year), 1 (1%) preschool child (3–5 years), 17 (30%) adults (18–64 years), and 10 (17%) elderly adults (>65 years). In 15 (26%) cases, age was not reported. Female patients were represented with 13 (22%) cases, and male patients were represented with 23 (40%) cases. Gender was not reported in 21 (37%) cases. Most of the cases were described in the United Kingdom (*n* = 13; 22%) followed by the United States of America and Turkey (*n* = 11; 19%) equally. Three cases of *Serratia marcescens* infections were described in Brazil, Germany, and Denmark. Two cases of SM infections were detected in Switzerland, Korea, Greece, and France. In Chile, Italy, Taiwan, Japan, and Argentina, one case of SM infection was detected. All patients were hospitalized. The longest duration of the study was reported in France (4 years). Characteristics of the included studies are shown in Table 1.

### 2.1. Site of S. marcescens Isolation

*S. marcescens* was, in most cases, isolated from blood (*n* = 26; 45%), followed by urine (*n* = 8; 14%), the cerebrospinal fluid (*n* = 7; 12%), and part of the tissue, e.g., the thyroid gland, leg, meninges, myocardium, fascia (*n* = 6; 10%). From corpus vitreum, *S. marcescens* was isolated in 6 cases (10%); from wound pus, SM was isolated in 5 cases (8%); from bronchoalveolar lavage and sputum, SM was isolated in 4 cases each (6%). Moreover, SM was isolated from bone (*n* = 3; 5%), peritoneal fluid (*n* = 2; 3%), as well as thoracic fluid and vaginal discharge (*n* = 1; 1%).

### 2.2. Method for S. marcescens Identification

The most common method for SM identification was colony morphology in combination with the biochemical system (*n* = 18; 31%). Only colony morphology was used in 14 cases (24%) and only the biochemical system was used in 11 cases (19%). The PCR assay was reported in 5 cases (8%); a combination of the biochemical system, MALDI-TOF mass spectrometry, and gene sequencing was used in 4 cases (6%); colony morphology and gene sequencing was used in 2 cases (3%).

The biochemical system and MALDI-TOF mass spectrometry, the biochemical systems, and gene sequencing, as well as MALDI-TOF mass spectrometry and gene sequencing were represented in one case.

### 2.3. Clinical Manifestation of S. marcescens Infection

In 22 patients (38%), signs of systemic infection were present; in other patients, signs were not reported (62%). The maximum level of C-reactive protein (CRP) was reported in six patients (10%). The maximum reported value of CRP was 405 mg/L. A count of white blood cells (WBC) was reported in 8 patients (14%) with a maximum level of about 43.300/mm^3^. In 12 cases (21%), the results from morphological diagnostics (e.g., ultrasound, radiography, CT) were obtained.

### 2.4. Sensitivity of S. marcescens to Antibiotics

**Figure 2:**
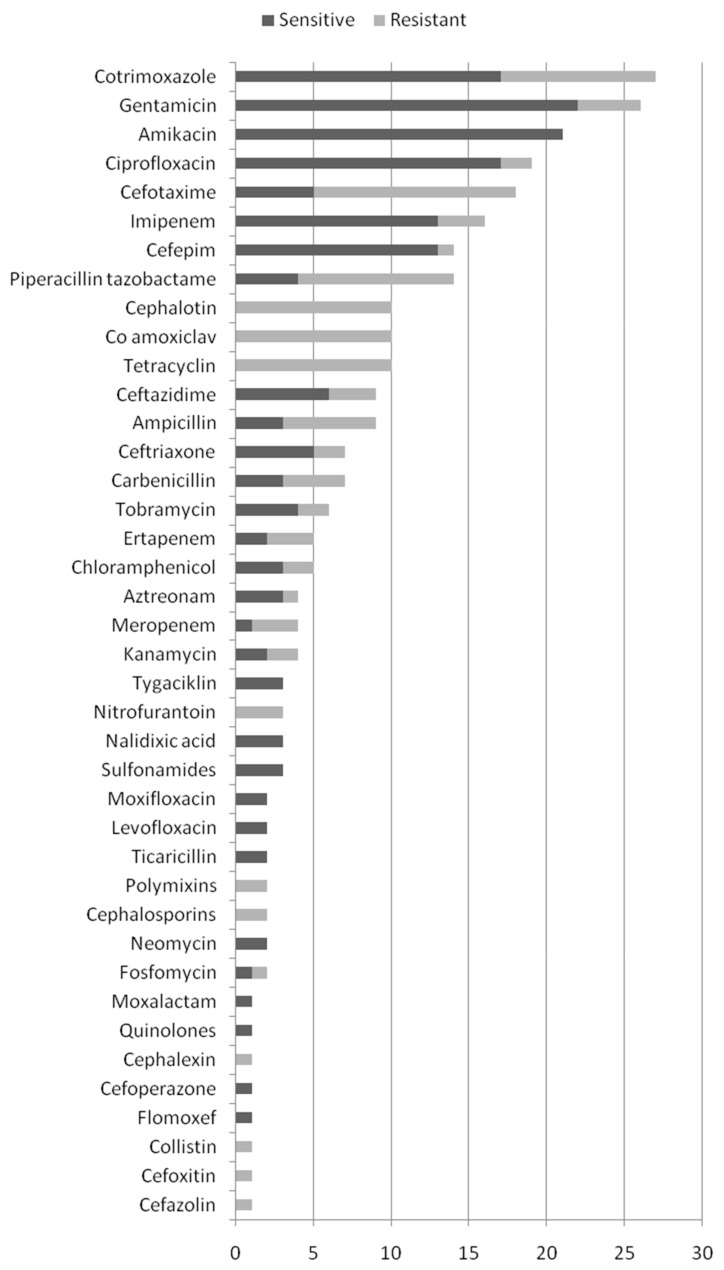
Susceptibility of *S. marcescens* to antibiotics.

In 42 patients, the susceptibility of *S. marcescens* to antibiotics was tested (73%) and the results are shown in Figure 2. In most cases, sensitivity was tested to cotrimoxazole (from 27 isolates, 10 showed resistance) followed by gentamicin (from 26 isolates, 3 showed resistance), as well as amikacin (from 21 isolates, none showed resistance). Isolates were also tested for ciprofloxacin (from 19 isolates, 2 showed resistance) and cefotaxime (from 18 isolates, 13 showed resistance). The isolates were tested on ampicillin, tetracycline, ceftazidime, cefalotin, ceftriaxone, imipenem, etc.

### 2.5. Antibiotics Used in the Treatment of Serratia marcescens Infection

**Figure 3:**
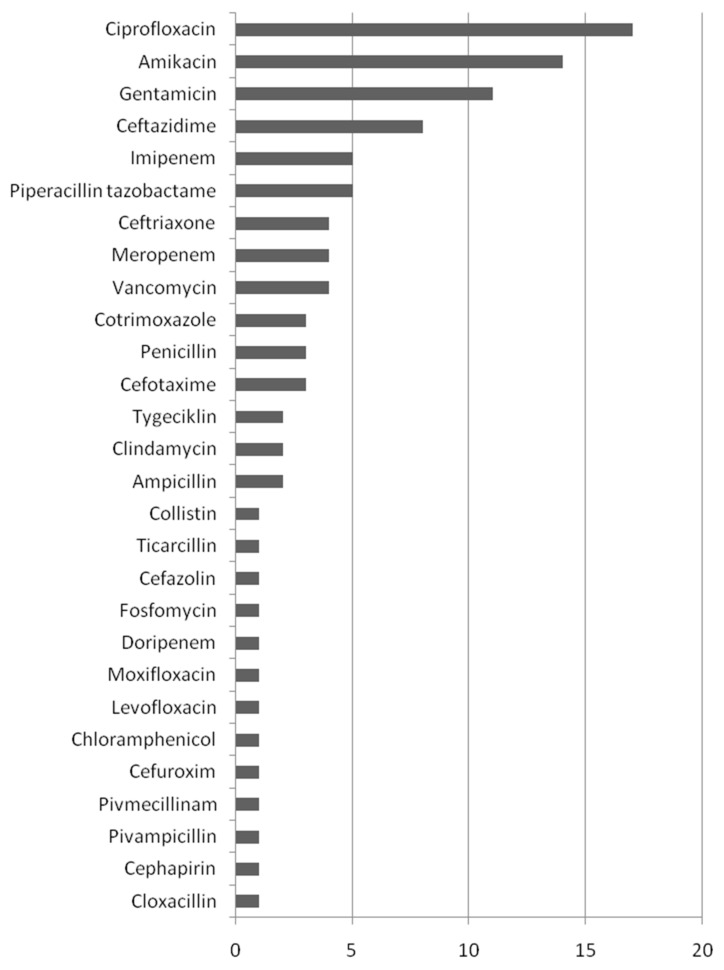
Antibiotics used for the treatment of *S. marcescens* infections.

In 15 cases (26%), antibiotic regimens were not reported. Only four patients were treated with one antibiotic. The most used antibiotic for the treatment of *Serratia marcescens* infection was ciprofloxacin (*n* = 17; 29%), followed by amikacin (*n* = 14; 24%) and gentamicin (*n* = 11; 19%). Antibiotic treatment is shown in Figure 3.

### 2.6. Treatment Outcomes

About half of the patients recovered completely (*n* = 29; 50%). Partial recovery was reported in five cases. In one case, the patient was cured of the infection but died from an underlying disease. Patients died from infections in 21 cases (31%). In one case, the outcome was not reported.

## 3. Discussion

Our results showed that *Serratia marcescens* may cause different types of infections in males and females, elderly patients, as well as newborns. SM was most frequently isolated from blood, urine, cerebrospinal fluid, and some tissues. Colony morphology in combination with the biochemical system (31%) was the most frequently used method for SM identification followed by only colony morphology and only the biochemical system. In most cases, isolates were susceptible to gentamicin, cotrimoxazole, amikacin, ciprofloxacin, and cefotaxime. Ciprofloxacin was the most frequently used antibiotic in the therapy of SM infections. The death rate was high (31%), which is what is worrying.

*Serratia marcescens* can produce a beta-lactamase, which influences resistance to the beta-lactam antibiotics and might complicate the therapy [4]. Carbapenem resistance in *S. marcescens* is associated with AmpC overexpression, Ambler class A production (KPC and SME), and B metallo-β-lactamase classes (MβLs; IMP, VIM). Serratia marcescens has carbapenem-hydrolyzing activity in the first production of KPC-2, and only in a few cases of KPC-3 and KPC-4 [19,23].

Aminoglycoside resistance is related to posttranscriptional methylation of 16S rRNA conferred by methyl transferases. To date, ten kinds of 16S rRNA methyl transferase genes (armA, rmtB, rmtA, rmtC, rmtE, rmtD, rmtF, rmtH, rmtG, and npmA) have been reported in Enterobacteriaceae. *S. marcescens* with rmtB, quinolone resistance genes, and various β-lactamase genes were also described [44]. Nowadays, gene sequencing is very popular for discovering antibiotic gene resistance and virulence factors of *Serratia marcescens* [45].

*S. marcescens* causes nosocomial infections in critically ill or immunocompromised patients, mostly in departments such as intensive care units [46]. SM can be found in soil, water, animals, plants, and insects [46]. There was an inverse relationship between virulence and the quantity of pigment (prodigiosin) produced [47]. In intensive care units, the most common reservoirs for infections are air-conditioning systems, washbasins, tap water, bronchoscopes, laryngoscopes, nebulizers, ventilation equipment, injectable solutions, liquid soap dispensers, etc. [46]. These are the reasons for the large number of hospital-related *S. marcescens* outbreaks [46].

There is no official therapy for SM infections. Results from our study showed sensitivity to gentamicin, cotrimoxazole, amikacin, ciprofloxacin, and cefotaxime. Sometimes gentamicin alone was the treatment of choice for SM infections but now it is more often used in combination with third-generation cephalosporins (e.g., cefotaxime) [4]. Trimethoprim-sulfamethoxazole (cotrimoxazole) is the most effective, especially for urinary tract infections. According to the literature, amikacin is active against gentamicin-resistant *S. marcescens* [4]. SM has a natural resistance to penicillin and first- and second-generation cephalosporin, tetracycline, macrolide, nitrofurantoin, and colistin [48]. When we look at the overall results, we notice that the therapy has changed over time; in the past, more aminoglycosides were prescribed. Today, it is colistin, tigecycline, and carbapenem.

Ciprofloxacin was the most frequently used antibiotic (29%) followed by amikacin and gentamicin. In most cases, ciprofloxacin was effective. According to the literature, ciprofloxacin use for the treatment of serious infections should be avoided due to its ability to develop resistance [4]. Amikacin and gentamicin were also frequently used. According to our results, carbapenems were not often used. According to some of the literature studies, it should be the antibiotic of choice [4,11,49]. This could be the reason for the mortality rate described in the literature according to our systematic review (31%). Third-generation cephalosporins (often combined with aminoglycosides) are the treatments of choice for uncomplicated infections. Fourth-generation cephalosporines are good treatment options where resistance to third-generation cephalosporins is evident [11]. They are active against AmpC chromosomal b-lactamase-producing strains as well as the treatment of ESBL-positive isolates [4]. According to the literature, piperacillin–tazobactam could also be an option for treating SM infections [50].

For the treatment of *Serratia marcescens* infections, in most cases, more than one antibiotic should be used, especially because of its ability to develop resistance [4]. Infections caused by SM could be fatal (according to our results in 31% of cases). According to the literature, the mortality rate is from 0% to 45% [4,51]. For this reason, infections caused by SM should be treated on time with the appropriate antibiotics.

The limitation of our systematic review involves the relatively small number of studies included. The second limitation was the reliability of the colony’s morphological identification as well as the biochemical identification of agents. More reliable methods, such as MALDI TOF or gene sequencing, were represented in smaller percentages of cases. Moreover, many cases (more than 50%) had high reporting biases, which may call into question the relevance of certain conclusions.

## 4. Materials and Methods

### 4.1. Data Sources

We registered this systematic review on the PROSPERO registry of systematic reviews–meta-analyses before we started our research (registration number CRD42022323159) [52].

An electronic database and collections of journals and books at the University Library, University of Kragujevac, Kragujevac, Serbia, were the bases for our search strategy in the identification of studies. The online searches of published studies were implemented via MEDLINE, the Cochrane Central Register of Controlled Trials, EBSCO, Scopus, Google Scholar, SCIndex, and the registry of clinical studies of human participants (ClinicalTrials.gov). Electronic databases were searched independently by three investigators: RZZ, MZ, and PC. The most extensive search strategy was made by RZZ on the MEDLINE database, i.e., ((Serratia Marcescens) AND (infection OR infections OR sepsis OR bacteremia OR pneumonia OR meningitis OR endocarditis OR osteomyelitis OR abscess OR peritonitis OR pericarditis OR pancreatitis OR pyelitis OR myocarditis OR arthritis OR bursitis OR endophthalmitis OR pyelonephritis OR septicemia OR meningoencephalitis OR urinary OR fasciitis) AND (case OR report OR series OR cohort OR observational OR case-control OR cross-sectional OR clinical trial) NOT (dermatitis OR keratitis OR ulcer OR scleritis OR conjunctivitis OR in vitro).

### 4.2. Participants and Study Eligibility Criteria

The inclusion criteria were as follows: type of study-case reports, case series, observational study, as well as clinical trial; characteristics of the participants—any age and gender with isolated *Serratia marcescens* as the only microorganism in the bodily fluids and tissues, identified by the following diagnostic methods: colony morphology, API, Vitek 2, or BD Phoenix biochemical systems, as well as matrix-assisted laser absorption ionization-time-of-flight mass spectrometry (MALDI-TOF MS) and specific PCR for *S. marcescens*. Exclusion criteria were as follows: review articles; cases of *S. marcescens* with non-human species; studies with incomplete dates. Papers were first assessed based on the titles and abstracts for eligibility, and then the full texts of the papers were read and analyzed. If all authors (RZZ, MZ, PC, MS, NZ, MS, MV, JN, VN, TV, JJ, DP, and SJ) agreed that the manuscript satisfied the inclusion criteria, it was further processed. In cases where the reviewers had different opinions about the eligibility of a study, the matter was resolved by the senior author (RZZ).

### 4.3. Interventions

The data from the studies included in the review were extracted to an Excel table with the following columns: publication ID, report ID, review author initials, design of the study, duration of the study, risk of bias, number of patients, age of patients, gender of patients, country, site of SM isolation, sampling method, method of SM identification, the maximal level of C-reactive protein, the maximal level of white blood cells, morphological diagnostics which confirmed invasive infection, presence of clinical sign of infection, antibiotics used, the outcome of antibiotic treatment, the resistance rate of SM to antibiotics.

The data were extracted by three investigators (RZZ, MZ, and PC) and the final extraction table was conducted by RZZ.

### 4.4. Data Analysis

All three investigators assessed the risk of bias (RZZ, MZ, and PC). The following sources of bias were accessed: reporting bias and attrition bias. The reporting bias was evaluated by checking what percentage of the target outcome was reported. For attrition bias, the number and percent of lost patients from the initial pool were estimated [53].

The categorical outcomes were the gender of patients, a method for SM identification, morphological diagnostics, outcomes of antibiotic treatment, adverse event rate, antibiotics used, as well as the resistance rate to SM. Continuous outcomes were the ages of patients, study duration, number of patients, the maximal level of C-reactive protein, and the maximal level of white blood cells.

## 5. Conclusions

*Serratia marcescens* infections should be taken seriously, especially because the bacterium causes high mortality and can lead to a high degree of resistance to antibiotics. The treatment of SM infections should include carbapenems or aminoglycosides in combination with third-generation (eventually fourth-generation cephalosporins). Cotrimoxazole should be considered in cases of uncomplicated urinary infections.

## Figures and Tables

**Table 1:** Overview of the reported *Serratia marcescens* cases.

Publication ID	Study Design	Attrition/Reporting Bias	Age	Gender	Site of Isolation of SM	Morphological Diagnosis of Infection	AB Used for Therapy	Outcomes of AB Treatment
Altweg et al., 1989 [12]	Case series	Low/High	NR	NR	Blood	NR	NR	CR
Altweg et al., 1989 [12]	Case series	Low/High	NR	NR	Bronchoalveolar lavage sample	P	NR	CR
Blajchman et al., 1979 [13]	Case series	Low/High	NR	M	Blood	NR	NR	CR
Blajchman et al., 1979 [13]	Case series	Low/High	NR	F	Blood	NR	NR	Death
Cambell et al., 1992 [14]	Case series	Low/Low	Newborn	F	Blood and cerebrospinal fluid	M	IV ampicillin plus gentamycin; IV cefotaxime plus amikacin	CR
Cambell et al., 1992 [14]	Case series	Low/Low	Newborn	M	Blood and cerebrospinal fluid	M	IV vancomycin plus amikacin; IV ampicillin plus gentamycin; amikacin plus cefotaxime; ceftazidime plus intraventricular amikacin	CR
Castro-Moraga et al., 2021 [15]	Case report	Low/Low	3	M	Pus from bone	O	IV penicillin plus clindamycin plus amikacin; IV ciprofloxacin plus cloxacillin	CR
Cayo et al., 2017 [16]	Case report	Low/Low	59	F	Blood	NR	IV meropenem; IV piperacillin–tazobactam plus ciprofloxacin	CR
Conacher et al., 1988 [17]	Case report	Low/Low	49	M	Peritoneal fluid	Pe	IP ceftriaxone plus gentamycin; IP gentamycin plus piperacillin plus cotrimoxazole	Death
Cope et al., 2013 [18]	Case report	Low/Low	97	F	Legg fascia tissue	F	NR	Death
Da Silva et al., 2021 [19]	Case series	Low/High	57	M	Bronchoalveolar lavage sample and urine	P	IV imipenem–cilastatin; ciprofloxacin	Death
Da Silva et al., 2021 [19]	Case series	Low/High	52	M	Urine	NR	IV piperacillin–tazobactam plus cotrimoxazole; meropenem plus vancomycin; ciprofloxacin	Death
Esel et al., 2002 [20]	Case series	Low/High	NR	NR	Blood and wound pus	Md	IV ciprofloxacin plus amikacin	CR
Esel et al., 2002	Case series	Low/High	NR	NR	Blood and wound pus	Md	IV imipenem plus amikacin	Death
Esel et al., 2002	Case series	Low/High	NR	NR	Blood and wound pus	Md	IV imipenem plus amikacin	CR
Esel et al., 2002	Case series	Low/High	NR	NR	Blood	Ed	IV ciprofloxacin plus amikacin	CR
Esel et al., 2002	Case series	Low/High	NR	NR	Blood	NR	IV ciprofloxacin plus amikacin	Death
Esel et al., 2002	Case series	Low/High	NR	NR	Blood	Ed	IV ciprofloxacin plus amikacin	Death
Esel et al., 2002	Case series	Low/High	NR	NR	Wound pus	WI	IV ciprofloxacin plus amikacin	CR
Esel et al., 2002	Case series	Low/High	NR	NR	Blood	NR	IV ciprofloxacin plus amikacin	CR
Esel et al., 2002	Case series	Low/High	NR	NR	Thoracic fluid	Md	IV ciprofloxacin plus amikacin	CR
Esel et al., 2002	Case series	Low/High	NR	NR	Wound pus	Md	IV ciprofloxacin plus amikacin	Death
Esmelizadeh et al., 2015 [21]	Case series	Low/Low	31	F	Urine and cerebrospinal fluid	M	IV meropenem plus ceftazidime	Death
Esmelizadeh et al., 2015	Case series	Low/Low	39	F	Wound pus and blood	WI	IV ceftriaxone plus ciprofloxacin; piperacillin–tazobactam; ciprofloxacin plus meropenem	CR
Gammon et al., 1980 [22]	Case report	Low/High	60	F	Urine and corpus vitreum	E	IV cefapirin; Tp gentamicin; amikacin	Death
Gona et al., 2017 [23]	Case report	High/High	NR	M	Bronchial lavage sample	P	NR	NR
Heltberg et al., 1993 [24]	Case series	Low/High	71	M	Blood	NR	IV penicillin G	CR
Heltberg et al., 1993	Case series	Low/High	52	M	Blood	NR	IV gentamicin plus PO ciprofloxacin	Died from malignancy
Heltberg et al., 1993	Case series	Low/High	73	M	Blood and urine	NR	PO ciprofloxacin; pivampicillin; pivmecillinam	Death
Huang et al., 2018 [25]	Case report	Low/High	57	M	Tissue culture	GL	NR	PR
Jonson et al., 1998 [26]	Case report	Low/Low	83	M	Urine, meninges, myocardium	M, My	IV cefuroxime	Death
Kufel et al., 2016 [27]	Case report	Low/Low	75	M	Urine and blood	NR	IV ceftriaxone; ciprofloxacin	CR
Lee et al., 2010 [28]	Case series	Low/High	53	M	Corpus vitreum	E	INV vancomycin and amikacin; vancomycin plus ceftazidime	PR
Lee et al., 2010	Case series	Low/Low	68	M	Corpus vitreum	E	INV vancomycin plus ceftazidime	PR
Lewis et al., 1982 [29]	Case series	Low/High	62	F	Cerebrospinal fluid	M	IV ceftazidime; gentamycin; chloramphenicol plus flucloxacillin	Death
Lewis et al., 1982	Case series	Low/High	25	M	Cerebrospinal fluid	M	IV ceftazidime plus gentamycin	CR
Liangpunsakul et al., 2001 [30]	Case report	Low/Low	25	M	Cerebrospinal fluid	M	IV clindamycin plus penicillin G plus ceftriaxone	Death
Memon et al., 2016 [31]	Case report	Low/Low	66	M	Corpus vitreum	E	IV levofloxacin plus Tp guttae ofloxacin plus Tp guttae ceftazidime	PR
Neonakis et al., 2014 [32]	Case report	High/High	67	NR	Peritoneal fluid and urine and bronchoalveolar sample	P	IV moxifloxacin plus tigecycline	CR
Paquin et al., 2021 [33]	Case report	Low/Low	61	M	Sternal collection	O	IV ceftriaxone plus gentamycin	Death
Rehman et al., 2012 [34]	Case report	Low/Low	54	F	Blood and part of the fascia	F	IV vancomycin plus piperacillin–tazobactam plus ciprofloxacin; doripenem	Death
Reichling et al., 1984 [35]	Case report	Low/Low	58	F	Thyroid abscess, blood, urine	T	IV moxalactam; cefotaxime	CR
Rieber et al., 2012 [36]	Case report	High/High	53	M	Blood and urine	NR	IV Imipenem	CR
Rodrigues et al., 2018 [37]	Case report	Low/High	NR	NR	Bone and soft tissue	O	IV fosfomycin plus ceftazidime	CR
Rowsey et al., 1982 [38]	Case report	Low/Low	83	F	Corpus vitreum	E	IC gentamicin and cephaloridine; gentamicin plus cephaloridine SB; gentamicin, cephaloridine a SB; IV gentamicin and cefazolin; IC gentamicin plus ticarcillin plus SC gentamicin, ticarcillin	CR
Rubens et al., 1980 [39]	Case report	Low/High	Newborn	M	Cerebrospinal fluid	M	IV gentamycin; amikacin	CR
Sevencan et al., 2018 [40]	Case report	Low/Low	89	M	Wound pus		IV ciprofloxacin	CR
Shimizu et al., 2003 [41]	Case report	Low/High	26	F	Blood, urine, and vaginal discharge	Ch	IV cefotiam; ceftazidime plus imipenem/cilastatin	PR
Smith et al., 1984 [42]	Case series	Low/High	28 weeks	NR	Blood	NR	NR	CR
Smith et al., 1984	Case series	Low/High	31 weeks	NR	Blood	NR	NR	Death
Smith et al., 1984	Case series	Low/High	28 weeks	NR	Blood	NR	NR	Death
Smith et al., 1984	Case series	Low/High	26 weeks	NR	Blood	NR	NR	Death
Smith et al., 1984	Case series	Low/High	30 weeks	NR	Sputum	P	NR	CR
Smith et al., 1984	Case series	Low/High	28 weeks	NR	Sputum	P	NR	CR
Smith et al., 1984	Case series	Low/High	28 weeks	NR	Sputum	P	NR	CR
Smith et al., 1984	Case series	Low/High	34 weeks	M	Sputum	P	NR	Death
Tsakris et al., 2010 [43]	Case report	Low/High	77	F	Bronchial lavage sample	P	IV tigecycline plus IH colistin	CR

Abbreviations: IV—intravenous, PO—per os, NR—not reported, CR—completely recovery, PR—partially recovery, M—male, F—female, AB—antibiotics, P—pneumonia, Ch—chorioamnionitis, M—meningitis, E—endophthalmitis, O—osteomyelitis, T-thyroiditis, Pe—peritonitis, F—fasciitis, My—myocarditis, Gl—gangrenous leg, Md—mediastinitis, WI—wound infection, Ed—endocarditis, IP—intraperitoneal, Tp—topica, INV—intravitreal, IC—intracamerally, SC— subconjunctivally, IH—inhaled.

## Data Availability

There are no available data.
